# Cost-effectiveness analysis of pembrolizumab plus chemotherapy versus chemotherapy in untreated advanced pleural mesothelioma in the Chinese healthcare system

**DOI:** 10.3389/fphar.2024.1402423

**Published:** 2025-01-07

**Authors:** Wenwang Lang, Yulong He, Changchun Hou, Hua Li, Qinling Jiang, Liuyong Mei

**Affiliations:** ^1^ Department of Pharmacy, Nanxishan Hospital of Guangxi Zhuang Autonomous Region, Guilin, China; ^2^ Department of Oncology, Nanxishan Hospital of Guangxi Zhuang Autonomous Region, Guilin, China; ^3^ Department of Pulmonary and Critical Care Medicine, Nanxishan Hospital of Guangxi Zhuang Autonomous Region, Guilin, China

**Keywords:** cost-effectiveness, pleural mesothelioma, non-epithelioid, pembrolizumab, markov mode

## Abstract

**Objective:**

The combination of pembrolizumab and chemotherapy has demonstrated notable clinical advantages in improving overall survival than chemotherapy alone for patients with untreated advanced pleural mesothelioma. The purpose of this study was to assess its cost-effectiveness.

**Materials and methods:**

A Markov state-transition model was constructed using data from the IND227 phase 3 randomized clinical trial. Utility values for health states were taken from the IND227 trial, and direct medical costs were from the pertinent literature and local pricing data. Outcomes measured included quality-adjusted life years (QALYs), incremental cost-effectiveness ratio (ICER), incremental net health benefit (INHB), and incremental net monetary benefit (INMB). To manage the uncertainty in the model, both probabilistic sensitivity analysis (PSA) and one-way sensitivity analysis (OWSA) were used.

**Results:**

In the base-case analysis, pembrolizumab plus chemotherapy resulted in an incremental gain of 0.23 QALYs at an additional cost of $18,199.63, resulting in an ICER of $80,557.23/QALY. This was not favorable compared to China’s willingness-to-pay (WTP) threshold of $38,042.49/QALY, with an INHB of −0.25 QALYs and an INMB of $-9,605.00. Subgroup analyses showed ICERs for pembrolizumab plus chemotherapy versus chemotherapy of $33,917.61 and $99,536.73 in non-epithelioid and epithelioid patients, respectively. PSA indicated probabilities of cost-effectiveness for pembrolizumab plus chemotherapy at 0.55%, 69.41%, and 0.14% for the entire population and the non-epithelioid and epithelioid subgroups, respectively.

**Conclusion:**

In the Chinese healthcare system, the combination of pembrolizumab and chemotherapy did not prove to be more cost-effective than chemotherapy alone as an initial treatment for untreated advanced pleural mesothelioma, with the exception of patients who have non-epithelioid histology.

## Introduction

In 2020, mesothelioma, a rare but highly aggressive form of cancer, resulted in 30,870 new cases and 26,278 deaths globally (Siegel et al., 2021; Sung et al., 2021). The predominant type of this cancer, malignant pleural mesothelioma (MPM), is often identified in advanced stages. MPM is classified into three histologic categories: epithelioid, sarcomatoid, and biphasic (Yap et al., 2017). For those with inoperable MPM, the preferred treatment approach is a chemotherapy combination of pemetrex- and platinum-based drugs (Popat et al., 2022). Although the pemetrexed-cisplatin combo offers clinical advantages compared to cisplatin alone, the median overall survival (OS) remains limited to approximately 12.1 months (Vogelzang et al., 2003). Some mesotheliomas express programmed death-ligand 1 (PD-L1), with the PD-L1 and PD-L2 ligands being present in tumor cells and their microenvironment. Blocking the interaction between PD-L1 and the PD-1 receptor in T cells can promote cancer suppression. Monoclonal antibodies that target the PD-1/PD-L1 pathway are gaining attention as a potential treatment option (Mansfield et al., 2014; Chapel et al., 2019; Sahu et al., 2023). Recent multicenter, randomized phase III clinical trials have indicated that immune checkpoint inhibitors, especially in combination therapies, can improve OS in patients with malignant mesotheliomas, exceeding the results of standard treatments (Baas et al., 2021; Fennell et al., 2021).

Pembrolizumab, a humanized monoclonal IgG4 kappa antibody targeting human PD-1, attaches to PD-1 without activating Fc-receptors or complement, thus exhibiting no cytotoxic effects (Wang and Khan, 2023). In the IND227 clinical trial (Chu et al., 2023), integrating pembrolizumab, an inhibitor of PD-1, into the standard chemotherapy regimen of platinum-pemetrexed significantly improved treatment outcomes for patients with advanced pleural mesothelioma, as compared to chemotherapy alone, leading to a 21% decrease in mortality risk. This improvement was evident in most subgroups, regardless of PD-L1 expression, even with a higher incidence of salvage immunotherapy in the group receiving only chemotherapy. The overall survival results achieved with this treatment approach were on par with those seen in studies using nivolumab-ipilimumab (Baas et al., 2021). Additionally, progression-free survival (PFS) and objective response rates (ORR) were more favorable. The safety profile of this combination in pleural mesothelioma patients mirrored that of advanced cases of non-small cell lung cancer. Although the inclusion of pembrolizumab resulted in a higher rate of adverse events, it did not negatively impact quality of life as reported by patients.

During the CheckMate-743 trial, analysis of subgroups revealed that individuals with non-epithelioid MPM experienced more significant improvements in OS. Patients with sarcomatoid MPM, who typically face a grimmer prognosis and less effective responses to chemotherapy, benefited more substantially from the combined treatment with nivolumab and ipilimumab. Similarly, the IND227 trial demonstrated a more notable advantage in patients with non-epithelioid histology. This underscores the importance of immunotherapy in the treatment regimen for these patients, especially since chemotherapy alone tends to be less effective (National Comprehensive Cancer Network, 2024).

Although the combination of pembrolizumab and chemotherapy has shown greater clinical effectiveness than chemotherapy alone, the financial rationale for this new treatment approach has not been determined. This study was conducted to evaluate the cost-effectiveness of pembrolizumab plus chemotherapy versus chemotherapy alone as a first-line treatment for advanced pleural mesothelioma in China. Considering the different clinical outcomes for patients with epithelioid or non-epithelioid histology, this analysis also focused on assessing cost-effectiveness within these specific subgroups.

## Materials and methods

### Patients and intervention

This study adhered to the Consolidated Health Economic Evaluation Reporting Standards (CHEERS) checklist (Husereau et al., 2022). It targeted patients with advanced pleural mesothelioma deemed unsuitable for surgery and who had not undergone previous systemic therapy for their advanced condition. However, [neo] adjuvant chemotherapy was allowed more than a year before treatment. Eligibility criteria included an Eastern Cooperative Oncology Group performance status score of 0 or 1, measurable disease as defined by the Response Evaluation Criteria in Solid Tumours (RECIST) version 1.1, modified for use in pleural mesothelioma (mRECIST) and the provision of a tumor sample for correlative analysis. Exclusion criteria included patients with untreated CNS metastases, pneumonitis, current glucocorticoid treatment equivalent to more than 10 mg daily of prednisone (within 7 days before the first dose of study treatment), or those suffering from concurrent serious illnesses or other cancers.

Eligible patients were treated with intravenous cisplatin (75 mg/m^2^) and pemetrexed (500 mg/m^2^) every 3 weeks for up to six cycles. Those randomly assigned to the pembrolizumab group also received intravenous pembrolizumab (200 mg) every 3 weeks for up to 2 years. Patient evaluations were conducted before each cycle, 4 weeks after discontinuation, every 12 weeks until progression, and biannually until death. Imaging for both groups was scheduled every 6 weeks for the first three assessments, followed by 12-week intervals.

Subsequent treatment regimens were based on the Chinese expert consensus on diagnosing and treating malignant pleural mesothelioma (2023 edition) (Wang et al., 2023), the IND227 trial, and clinical practice. In the pembrolizumab plus chemotherapy group, patients received bevacizumab plus gemcitabine plus carboplatin, while the chemotherapy group received nivolumab. The doses of chemotherapy agents were calculated using a reference patient weighing 65.00 kg with a height of 1.64 m (body surface area of 1.72 m^2^). The current pembrolizumab and nivolumab patient assistance programs were considered for their effect on price reductions for these drugs.

The cost implications of adverse events (AEs) were determined based on data from the IND227 trial. In our analysis, only grade 3/4 AEs, classified as serious adverse events (SAEs), were considered, and this was restricted to those with incidence rates exceeding 5%. These SAEs included fatigue, anemia, thrombocytopenia, neutropenia, increased lipase levels, and hyperglycemia.

### Model structure

**FIGURE 1:**
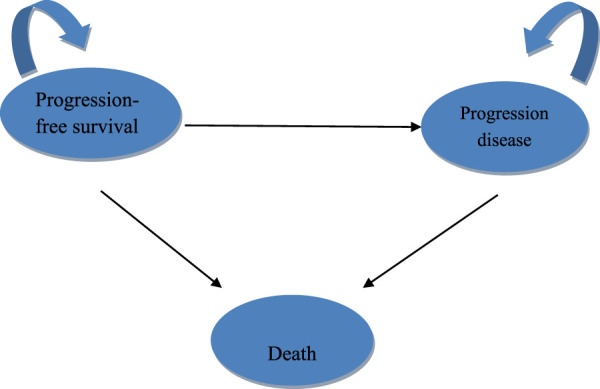
Model structure.

The development and outcome analysis of the model was conducted using TreeAge Pro 2022 software (Williamstown, MA, USA) and R software (version 4.2.3, Vienna, Austria). A three-state Markov model was implemented (Freitag et al., 2024; Wei et al., 2024), which comprised the following health states: progression-free survival (PFS), progressive disease (PD), and death (Figure 1).

All subjects entered the model in the PFS state and were either treated with chemotherapy alone or a combination of pembrolizumab and chemotherapy until disease progression or the emergence of intolerable toxicity. Patients whose disease advanced were transitioned to the PD state, where they received additional therapies following the discontinuation of pembrolizumab or placebo. The fraction of patients in the PD state was determined based on the area under the OS curve, the proportion of surviving patients, the proportion of patients with PFS, and the difference between the OS and PFS curves.

The simulation was performed over a period of 10 years, thereby encompassing over 99% of the mortality events in both treatment cohorts. The analysis was conducted from the vantage point of the Chinese healthcare systems, integrating all costs associated with healthcare services.

### Outcomes

The primary outcomes measured were quality-adjusted life years (QALYs) and costs. To account for the value of time and adjust future costs and benefits, a 5% annual discount rate was used, as recommended by the World Health Organization (WHO) guidelines for pharmacoeconomic evaluations (Chinese Pharmaceutical Association, 2024). All costs were adjusted for 2023 prices using the local consumer price index and converted to US dollars at an exchange rate of $1 = ¥7.0467. The analysis included a cost-effectiveness evaluation, expressed in terms of incremental cost-effectiveness ratios (ICERs). The ICER was calculated as follows: ICER = [Cost (pembrolizumab plus chemotherapy) - Cost (chemotherapy)]/[QALY (pembrolizumab plus chemotherapy) - QALY (chemotherapy)]. The willingness-to-pay (WTP) threshold was three times China’s *per capita* gross domestic product (GDP) in 2023, $38,042.49, according to WHO recommendations. Furthermore, this study incorporated assessments of the incremental net health benefit (INHB) and the incremental net monetary benefit (INMB). These values were computed using the following equations: INHB (λ) = (μE1 - μE0) - (μC1 - μC0)/λ = ΔE - ΔC/λ and INMB (λ) = (μE1 - μE0) × λ - (μC1 - μC0) = ΔE × λ - ΔC. In these equations, μCi and μEi represent the costs and utility associated with pembrolizumab plus chemotherapy (i = 1) or chemotherapy alone (i = 0), respectively, with λ denoting the WTP threshold.

### Clinical data input

Survival curves for OS and FS in the IND227 trial were constructed using the method proposed by Guyot et al. (2012). Time-to-event data points for the Kaplan-Meier survival curves for OS and PFS were extracted using GetData Graph Digitizer version 2.26 (www.getdata.graph.digitizer.com). These data points were then applied to fit a range of parametric survival models, including Exponential, Weibull, WeibullPH, Gamma, Log-normal, Gompertz, Generalized Gamma, and Log-logistic distributions.

**FIGURE 2:**
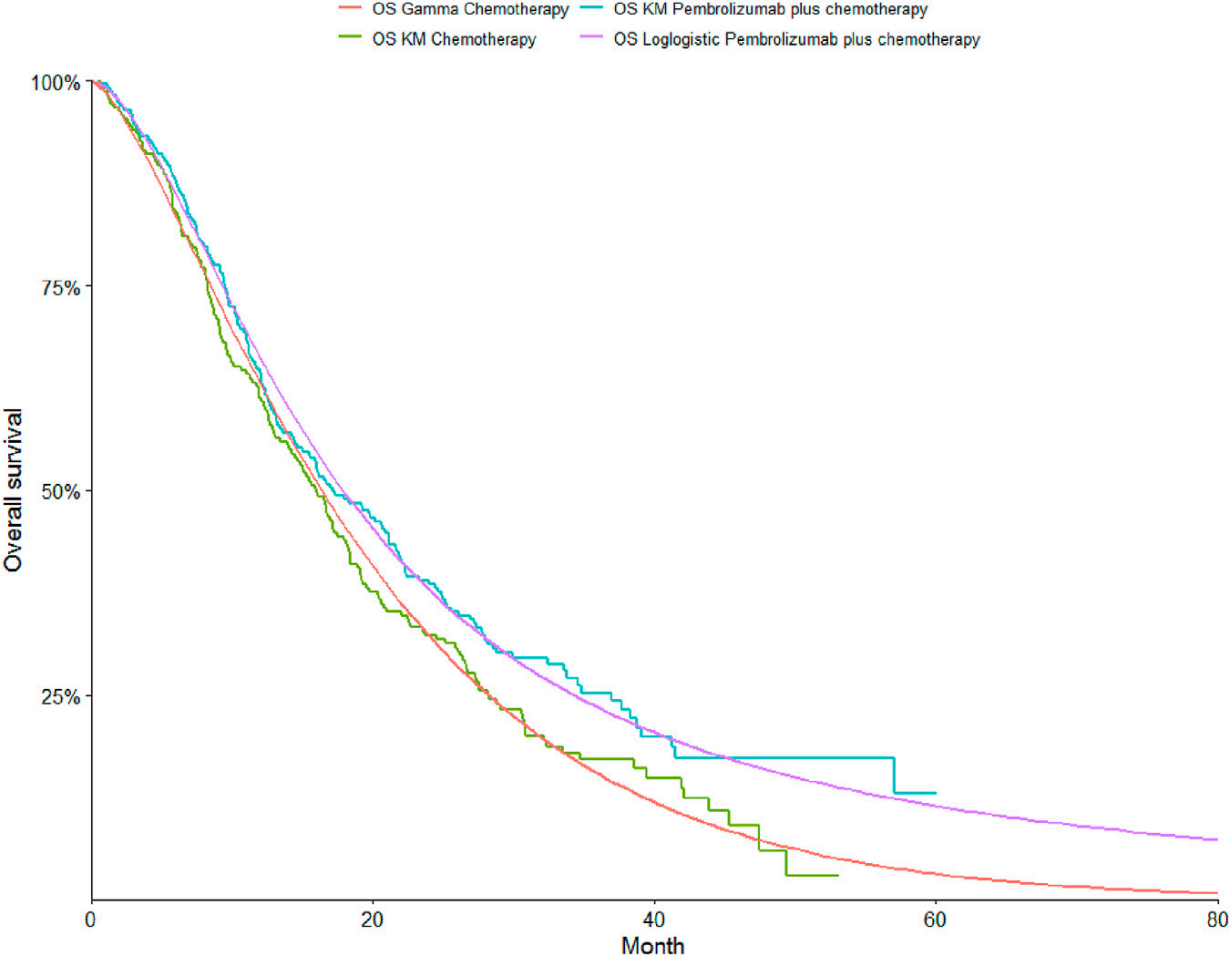
The Kaplan-Meier overall survival curves.

**FIGURE 3:**
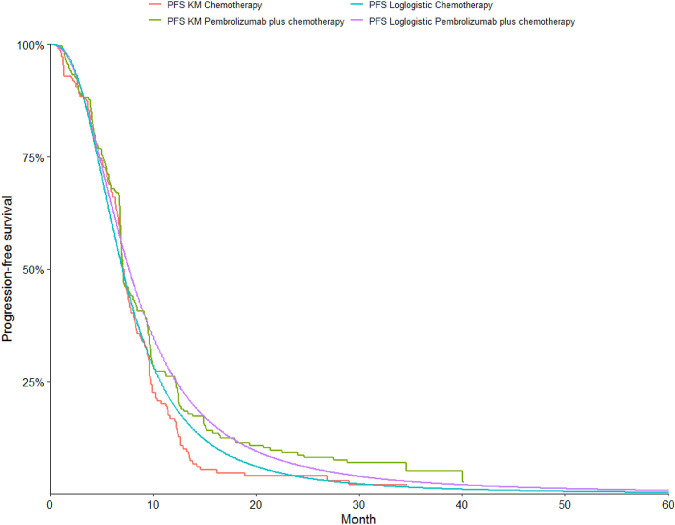
The Kaplan-Meier progression-free survival curves.

**FIGURE 4:**
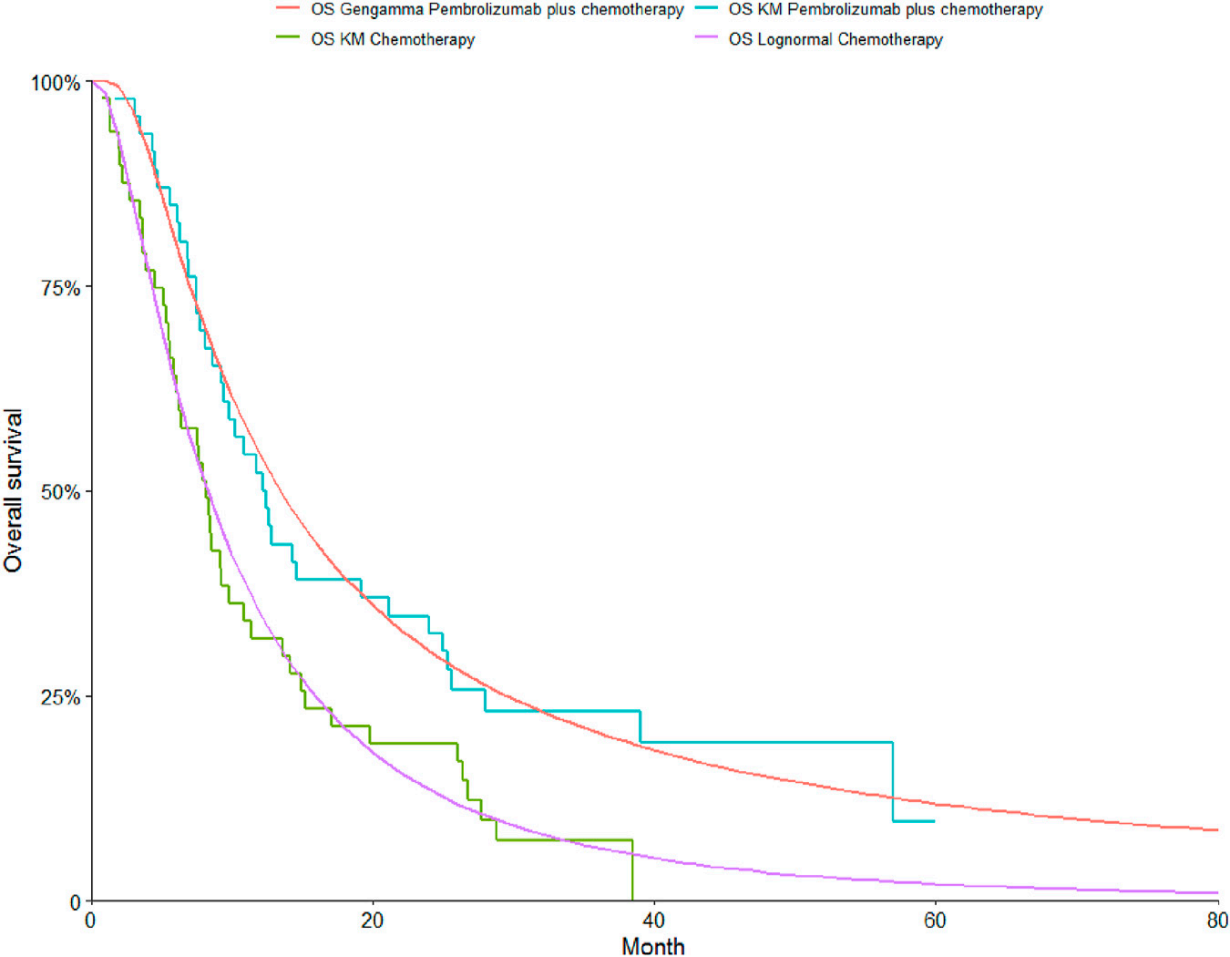
The Kaplan-Meier overall survival curves in patients with non-epithelioid.

**FIGURE 5:**
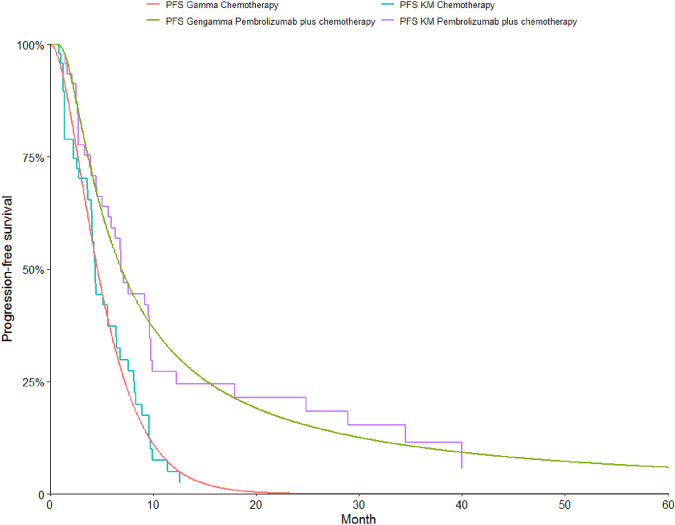
The Kaplan-Meier progression-free survival curves in patients with non-epithelioid.

**FIGURE 6:**
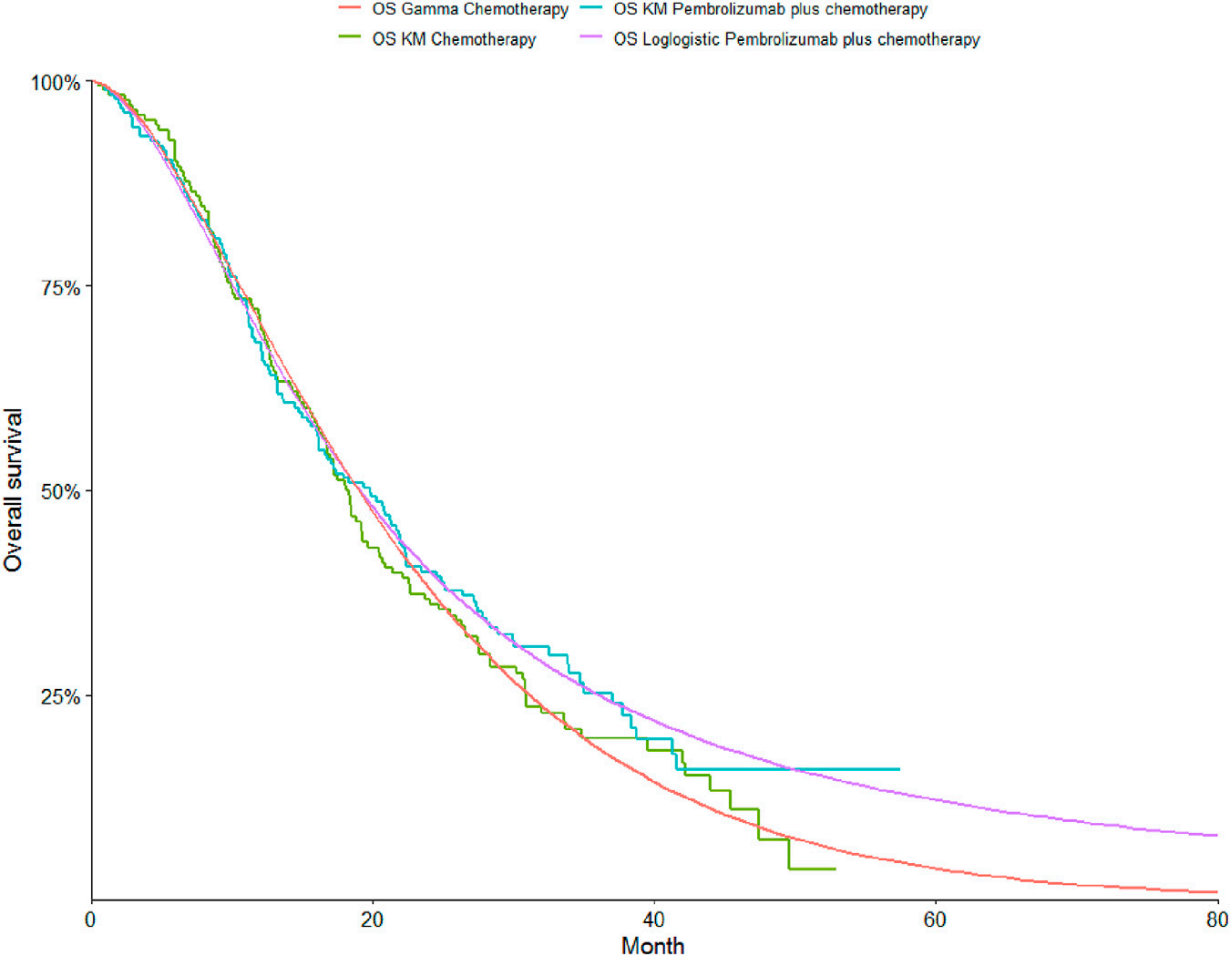
The Kaplan-Meier overall survival curves in patients with epithelioid.

**FIGURE 7:**
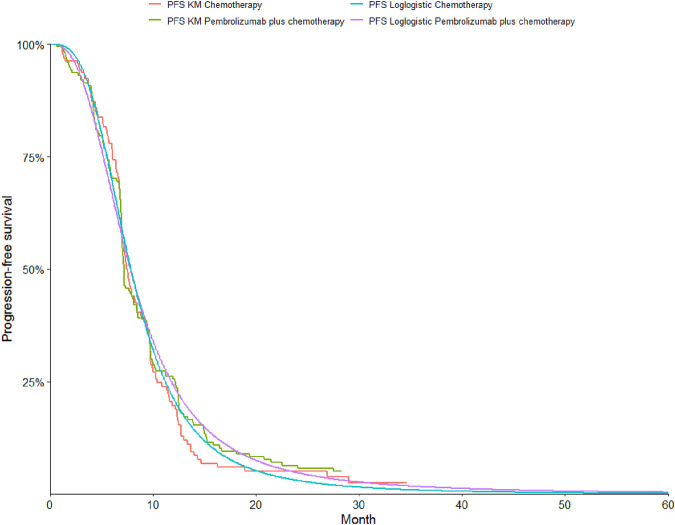
The Kaplan-Meier progression-free survival curves in patients with epithelioid.

To determine the most suitable survival curves for PFS and OS, the fits were evaluated using both the Akaike Information Criterion (AIC) and the Bayesian Information Criterion (BIC), along with a visual review of the results (as presented in Tables 3-5). The estimated shape parameters (g) and scale parameters (l) for these curves are outlined in Table 1. Additionally, extensive details on long-term survival data can be found in Table 1 and Figures 2–7.

**TABLE 1:** Model parameters: baseline values, ranges, and distributions for the sensitivity analysis.

Parameters		Baseline value	Range		Distribution	References
			Minimum	Maximum		
*Survival model for OS*						
Pembrolizumab plus chemotherapy		Shape = 1.6860 Scale = 17.8960			Loglogistic	Chu et al. (2023)
Chemotherapy		Shape = 1.6860 Rate = 0.0743			Gamma	Chu et al. (2023)
*Survival model for PFS*						
Pembrolizumab plus chemotherapy		Shape = 2.3470 Scale = 7.6780			Loglogistic	Chu et al. (2023)
Pembrolizumab plus chemotherapy		Shape = 2.6100 Scale = 7.0350			Loglogistic	Chu et al. (2023)
*Survival model for OS (non-epithelioid)*						
Pembrolizumab plus chemotherapy		mu = 2.3140 sigma = 0.9550 Q = −0.8350			Gengamma	Chu et al. (2023)
Chemotherapy		Meanlog = 2.114 Sdlog = 0.9690			Lognormal	Chu et al. (2023)
*Survival model for PFS (non-epithelioid)*						
Pembrolizumab plus chemotherapy		mu = 1.6226 sigma = 0.9186 Q = −0.9420			Gengamma	Chu et al. (2023)
Chemotherapy		Shape = 2.1439 Rate = 0.3955			Gamma	Chu et al. (2023)
*Survival model for OS (epithelioid)*						
Pembrolizumab plus chemotherapy		Shape = 1.7230 Scale = 19.0840			Loglogistic	Chu et al. (2023)
Chemotherapy		Shape = 1.8344 Rate = 0.0796			Gamma	Chu et al. (2023)
*Survival model for PFS (epithelioid)*						
Pembrolizumab plus chemotherapy		Shape = 2.6670 Scale = 7.8090			Loglogistic	Chu et al. (2023)
Chemotherapy		Shape = 3.1000 Scale = 7.8590			Loglogistic	Chu et al. (2023)
*Drug cost, $/per cycle*						
Cost of pembrolizumab		4654.66	3723.73	5585.59	Gamma	Local charge
Cost of pemetrexed		143.53	114.82	172.24	Gamma	Local charge
Cost of cisplatin		35.03	28.02	42.04	Gamma	Local charge
Cost of nivolumab		3279.69	2623.75	3935.63	Gamma	Local charge
Cost of bevacizumab		496.69	397.35	596.03	Gamma	Local charge
Cost of gemcitabine		153.21	122.57	183.85	Gamma	Local charge
Cost of carboplatin		43.94	35.15	52.73	Gamma	Local charge
Cost of the laboratory test		120.96	96.77	145.15	Gamma	Li et al. (2021), Lin et al. (2021)
Chest and abdominal enhanced CT		268.88	215.10	322.66	Gamma	Li et al. (2021)
Cost of end-of-life		1,460.30	1168.24	1752.36	Gamma	Wu et al. (2014)
Cost of drug administration	Preventive medication per intravenous administration	93.93	75.14	112.72	Gamma	Liu et al. (2023a), Zhu et al. (2023)
	Infusion fee	1.86	1.49	2.23	Gamma	Liu et al. (2023a), Zhu et al. (2023)
	Preventive medication	39.14	31.31	46.97	Gamma	Liu et al. (2023a), Zhu et al. (2023)
Utility of PFS		0.706	0.565	0.847	Beta	Morris et al. (2014), Yang et al. (2022a), Zhu et al. (2022)
Utility of PD		0.565	0.452	0.678	Beta	Morris et al. (2014), Yang et al. (2022a), Zhu et al. (2022)
Discount rate		5%	0	8.00%	Beta	
BMI/m2		1.72				
Weight/kg		65.00				
$1 = ¥7.0467						

OS: overall survival, PFS: progression-free survival, PD: progression disease, BMI: body mass index.

**TABLE 2:** AEs: baseline values, ranges, and distributions for the sensitivity analysis.

Parameters	Baseline value	Range		Distribution	References
		Minimum	Maximum		
*Cost of AEs, $*					
Neutropenia	411.93	329.54	494.32	Gamma	Wu et al. (2020)
Anemia	921.10	736.88	1105.32	Gamma	Wu et al. (2020)
Thrombocytopenia	1523.82	1219.06	1828.58	Gamma	Liu et al. (2023b)
Lipase increase	44.30	35.44	53.16	Gamma	Shu et al. (2022)
Fatigue	103.00	82.40	123.60	Gamma	Wu et al. (2020)
Hyperglycemia	361.91	289.53	434.29	Gamma	Local charge
*Disutility estimates*					
Neutropenia	0.09	0.07	0.11	Beta	Scherpereel et al. (2022)
Anemia	0.12	0.01	0.14	Beta	Scherpereel et al. (2022)
Thrombocytopenia	0.11	0.09	0.13	Beta	Liu et al. (2023b)
Lipase increase	0.47	0.38	0.56	Beta	Zhan et al. (2017)
Fatigue	0.29	0.23	0.35	Beta	Scherpereel et al. (2022)
Hyperglycemia	0.20	0.16	0.24	Beta	Yang et al. (2022b)
*Risk for main AEs in Pembrolizumab plus Chemotherapy group*					
Fatigue	6.76%	5.41%	8.11%	Beta	Chu et al. (2023)
Anemia	23.87%	19.10%	28.64%	Beta	Chu et al. (2023)
Thrombocytopenia	10.81%	8.65%	12.97%	Beta	Chu et al. (2023)
Neutropenia	31.98%	25.58%	38.38%	Beta	Chu et al. (2023)
Lipase increase	7.21%	5.77%	8.65%	Beta	Chu et al. (2023)
Hyperglycemia	6.31%	5.05%	7.57%	Beta	Chu et al. (2023)
*Risk for main AEs in Placebo plus Chemotherapy group*					
Fatigue	5.69%	4.55%	6.83%	Beta	Chu et al. (2023)
Anemia	13.27%	10.62%	15.92%	Beta	Chu et al. (2023)
Thrombocytopenia	4.74%	3.79%	5.69%	Beta	Chu et al. (2023)
Neutropenia	15.77%	12.62%	18.92%	Beta	Chu et al. (2023)
Lipase increase	2.37%	1.90%	2.84%	Beta	Chu et al. (2023)
Hyperglycemia	4.27%	3.42%	5.12%	Beta	Chu et al. (2023)

AE: adverse event.

**TABLE 3:** The Akaike information criteria (AIC) and Bayesian information criteria (BIC).

Type of distribution	Pembrolizumab plus chemotherapy (OS)		Chemotherapy (OS)		Pembrolizumab plus chemotherapy (PFS)		Chemotherapy (PFS)	
	AIC	BIC	AIC	BIC	AIC	BIC	AIC	BIC
Exponential	1431.653	1435.055	1429.300	1432.684	1308.108	1311.510	1100.305	1103.690
Gamma	1421.216	1428.022	1413.456	1420.225	1268.875	1275.680	1035.641	1042.410
Generalized gamma	1415.594	1425.803	1414.472	1424.626	1246.798	1257.006	1036.040	1046.193
Gompertz	1432.687	1439.492	1423.964	1430.733	1308.809	1315.614	1086.945	1086.945
Weibull	1424.758	1431.563	1415.618	1422.387	1284.595	1291.401	1047.209	1053.978
WeibullPH'	1424.758	1431.563	1415.618	1422.387	1284.595	1291.401	1047.209	1053.978
Log-logistic	1413.186	1419.992	1415.068	1421.837	1235.957	1242.762	1027.145	1033.914
Lognormal	1414.039	1420.844	1419.843	1426.612	1245.162	1251.967	1042.747	1049.516

OS:overall survival; PFS:progression-free survival; AIC:akaike information criterion; BIC:bayesian information criterion.

**TABLE 4:** The Akaike information criteria (AIC) and Bayesian information criteria (BIC) (Non-epithelioid).

Type of distribution	Pembrolizumab plus chemotherapy (OS)		Chemotherapy (OS)		Pembrolizumab plus chemotherapy (PFS)		Chemotherapy (PFS)	
	AIC	BIC	AIC	BIC	AIC	BIC	AIC	BIC
Exponential	306.958	308.786	312.767	314.658	265.103	266.931	231.667	233.559
Gamma	307.368	311.025	312.331	316.115	266.485	270.142	221.418	225.201
Generalized gamma	299.505	304.991	310.847	316.523	256.308	261.794	223.416	229.091
Gompertz	308.476	312.133	314.700	318.484	265.738	269.395	224.038	227.821
Weibull	308.421	312.078	313.362	317.145	267.067	270.724	221.633	225.417
WeibullPH'	308.421	312.078	313.362	317.145	267.067	270.724	221.633	225.417
Log-logistic	301.180	304.837	309.621	313.405	258.511	262.168	225.361	229.145
Lognormal	300.089	303.747	308.849	312.632	257.536	261.193	222.887	226.671

OS:overall survival; PFS:progression-free survival; AIC:akaike information criterion; BIC:bayesian information criterion.

**TABLE 5:** The Akaike information criteria (AIC) and Bayesian information criteria (BIC) (Epithelioid).

Type of distribution	Pembrolizumab plus chemotherapy (OS)		Chemotherapy (OS)		Pembrolizumab plus chemotherapy (PFS)		Chemotherapy (PFS)	
	AIC	BIC	AIC	BIC	AIC	BIC	AIC	BIC
Exponential	1125.885	1129.056	1098.217	1101.347	1040.968	1044.139	848.991	852.121
Gamma	1115.624	1121.965	1075.219	1081.479	986.706	993.047	780.204	786.464
Generalized gamma	1115.416	1124.928	1077.030	1086.420	980.539	990.051	778.080	787.469
Gompertz	1125.007	1131.348	1087.322	1093.582	1031.932	1038.273	835.696	841.956
Weibull	1117.809	1124.150	1077.156	1083.416	1000.617	1006.958	796.299	802.558
WeibullPH'	1117.809	1124.150	1077.156	1083.416	1000.617	1006.958	796.299	802.558
Log-logistic	1112.592	1118.933	1075.521	1081.781	967.609	973.950	761.080	767.340
Lognormal	1115.779	1122.120	1083.387	1089.646	979.353	985.694	778.980	785.240

OS:overall survival; PFS:progression-free survival; AIC:akaike information criterion; BIC:bayesian information criterion.

### Cost input

This analysis was conducted from the perspective of the Chinese healthcare system, emphasizing direct costs. These include drug expenses, laboratory test fees, costs associated with enhanced chest and abdomen CT scans, preventive medications for each intravenous administration, end-of-life expenses, infusion fees per intravenous administration, subsequent treatment costs, and expenses related to managing grades 3 and 4 AEs. Drug costs were derived from national medical insurance negotiation prices and local charges, while other cost data were from previously published research studies and relevant literature.

The drug doses for the patients were determined using the IND227 study protocol. Treatment costs per cycle were determined based on dose schedules and the local price (Table 1) (Wu et al., 2014; Li et al., 2021; Lin et al., 2021; Liu L. et al., 2023; Zhu et al., 2023). The cost associated with each AE was calculated by multiplying the incidence rate of the AE by the per-occurrence cost of managing these events (Wu et al., 2020; Shu et al., 2022; Liu S. et al., 2023). We assumed that all AEs occurred during the first cycle of treatment. Table 2 presents the incidence rates for each AE in detail.

### Quality-of-life inputs

The health utility scores in our model were assigned on a scale ranging from death (0) to perfect health (1). Due to the unavailability of the European Quality of Life-5 Dimensions-5 Level (EQ-5D-5L) data from the IND227 trial, direct quality-of-life data could not be obtained. Consequently, the utility values were from the relevant published literature (Table 1). The utility values for PFS and PD were set at 0.706 and 0.565, respectively (Zhan et al., 2017; Scherpereel et al., 2022; Yang L. et al., 2022). AEs were considered to decrease health utility, a concept known as disutility (Morris et al., 2014; Yang J. et al., 2022; Zhu et al., 2022; Liu S. et al., 2023). This disutility associated with AEs was incorporated only in the first cycle of the model and was assumed to occur once every 3 weeks.

### Sensitivity analysis

Two sensitivity analyses were conducted to address the inherent uncertainty: one-way sensitivity analysis (OWSA) and probabilistic sensitivity analysis (PSA). In the OWSA, the parameter ranges were from the existing published literature, and the parameter variations were set to ±20% of the baseline values (Morris et al., 2014; Wu et al., 2014; Zhan et al., 2017; Wu et al., 2020; Li et al., 2021; Lin et al., 2021; Scherpereel et al., 2022; Shu et al., 2022; Yang J. et al., 2022; Yang L. et al., 2022; Zhu et al., 2022; Liu L. et al., 2023; Liu S. et al., 2023; Zhu et al., 2023). For PSA, the model parameters were altered simultaneously across 10,000 Monte Carlo simulations, allowing an evaluation of the probability that each intervention would be cost-effective in varying the WTP thresholds for an additional QALY. In these simulations, beta distributions were assigned to utility parameters, and gamma distributions were used for cost variables. The outcomes of this analysis are shown in a scatter plot and a cost-effectiveness acceptability curve.

### Subgroup analyses

In subgroup analyses, the cost-effectiveness of pembrolizumab plus chemotherapy versus chemotherapy alone as first-line treatment for untreated advanced pleural mesothelioma was assessed separately for patients with epithelioid and non-epithelioid histologies. This assessment used base-case analysis, OWSA, and PSA methods. Due to the lack of distinct data in the IND227 trial in the follow-up treatment plan, drug selection, and the occurrence of adverse reactions specific to the epithelioid and non-epithelioid subgroups, it was assumed that these factors were consistent with those observed in the general study population.

## Results

### Base-case analysis

During a 10-year analysis horizon, base-case findings revealed that the pembrolizumab plus chemotherapy group achieved an additional 0.99 QALYs at an incremental cost of $35,560.57. In contrast, the chemotherapy-only group gained 0.77 QALYs, with associated costs totaling $17,360.94. Comparative analysis between pembrolizumab plus chemotherapy and chemotherapy alone indicated a mean incremental effect of 0.23 QALYs and an additional cost of $18,199.63. This resulted in an ICER for pembrolizumab plus chemotherapy versus chemotherapy of $80,557.23 per QALY, as shown in Table 5. In particular, when evaluated against the China WTP cost-effectiveness threshold of $38,042.49 per QALY, pembrolizumab plus chemotherapy was not more cost-effective than chemotherapy. Additionally, at this WTP threshold, pembrolizumab plus chemotherapy, relative to chemotherapy, demonstrated an INHB of −0.25 QALYs and an INMB of $-9,605.00 (Table 5).

### Subgroup analysis

In the subgroup analyses, the ICER for pembrolizumab plus chemotherapy versus chemotherapy alone was $33,917.61 per QALY gained for patients with non-epithelioid histology and $99,536.73 per QALY for those with epithelioid histology. In particular, in the non-epithelioid subgroup, ICER was below the China WTP threshold of $38,042.49 per QALY, as shown in Table 6. Furthermore, the INHB associated with pembrolizumab plus chemotherapy was 0.05 QALYs for non-epithelioid patients and −0.29 QALYs for epithelioid patients. Regarding INMB, the values were $2,085.28 and $-11,127.42, respectively, at the WTP threshold of $38,042.49 per QALY compared to chemotherapy, as detailed in Table 6.

**TABLE 6:** The base case analysis.

Treatment	Cost	QALY	Incremental cost	Incremental QALY	INHB	INMB	ICER
Pembrolizumab plus chemotherapy	35560.57	0.99	18199.63	0.23	−0.25	−9605.00	80557.23
Chemotherapy	17360.94	0.77					
Pembrolizumab plus chemotherapy (Non-epithelioid)	29315.92	0.99	17138.29	0.51	0.05	2085.28	33917.61
Chemotherapy (Non-epithelioid)	12177.63	0.49					
Pembrolizumab plus chemotherapy (Epithelioid)	37397.91	1.03	18012.41	0.18	−0.29	−11127.42	99536.73
Chemotherapy (Epithelioid)	19385.50	0.85					

QALY: Quality-adjusted life year, ICER: Incremental cost-effectiveness ratio, INMB:the incremental net monetary benefits, INHB: the incremental net health benefits.

### Sensitivity analysis

**FIGURE 8:**
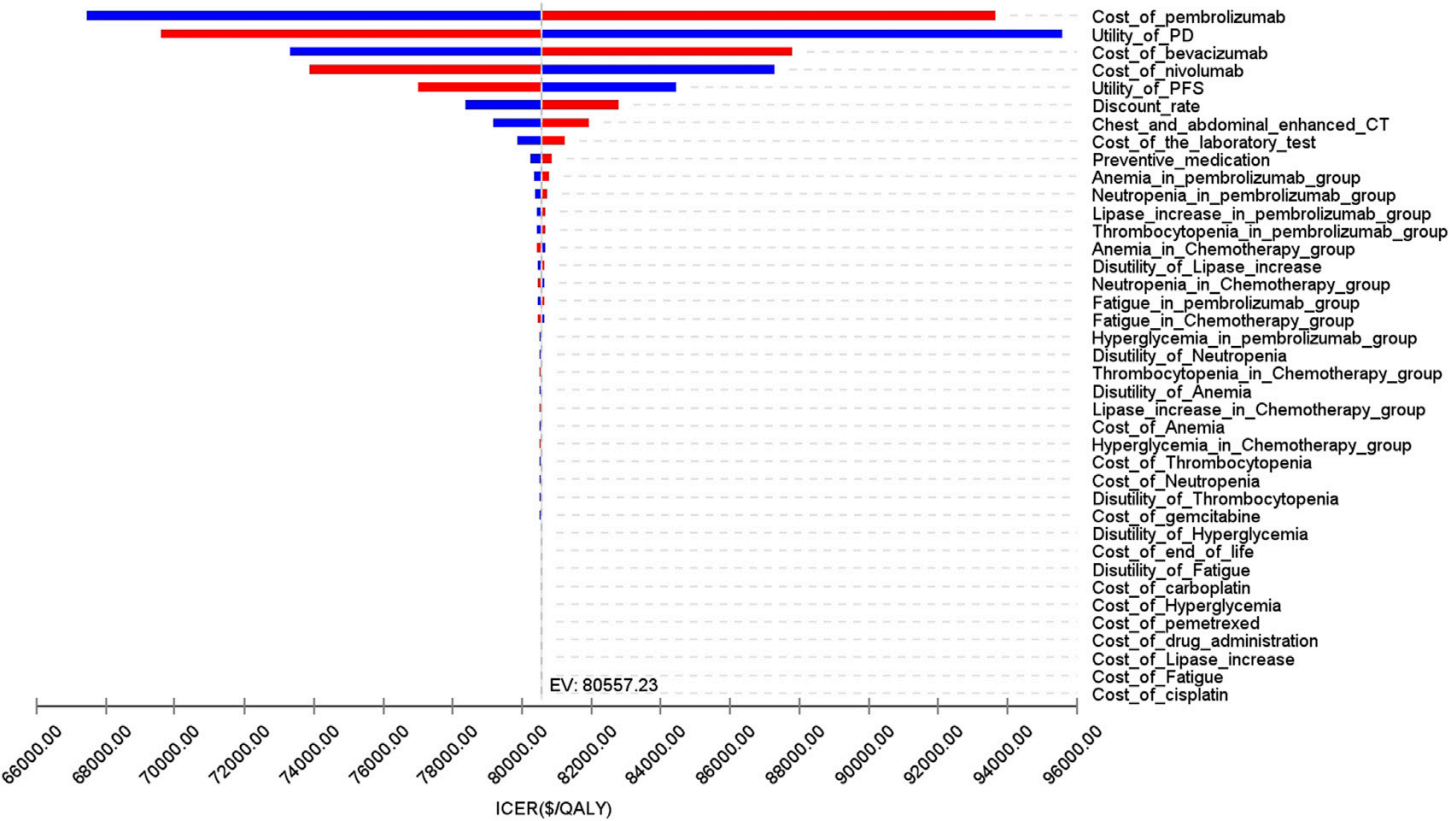
The tornado diagram of one-way sensitivity analysis.

**FIGURE 9:**
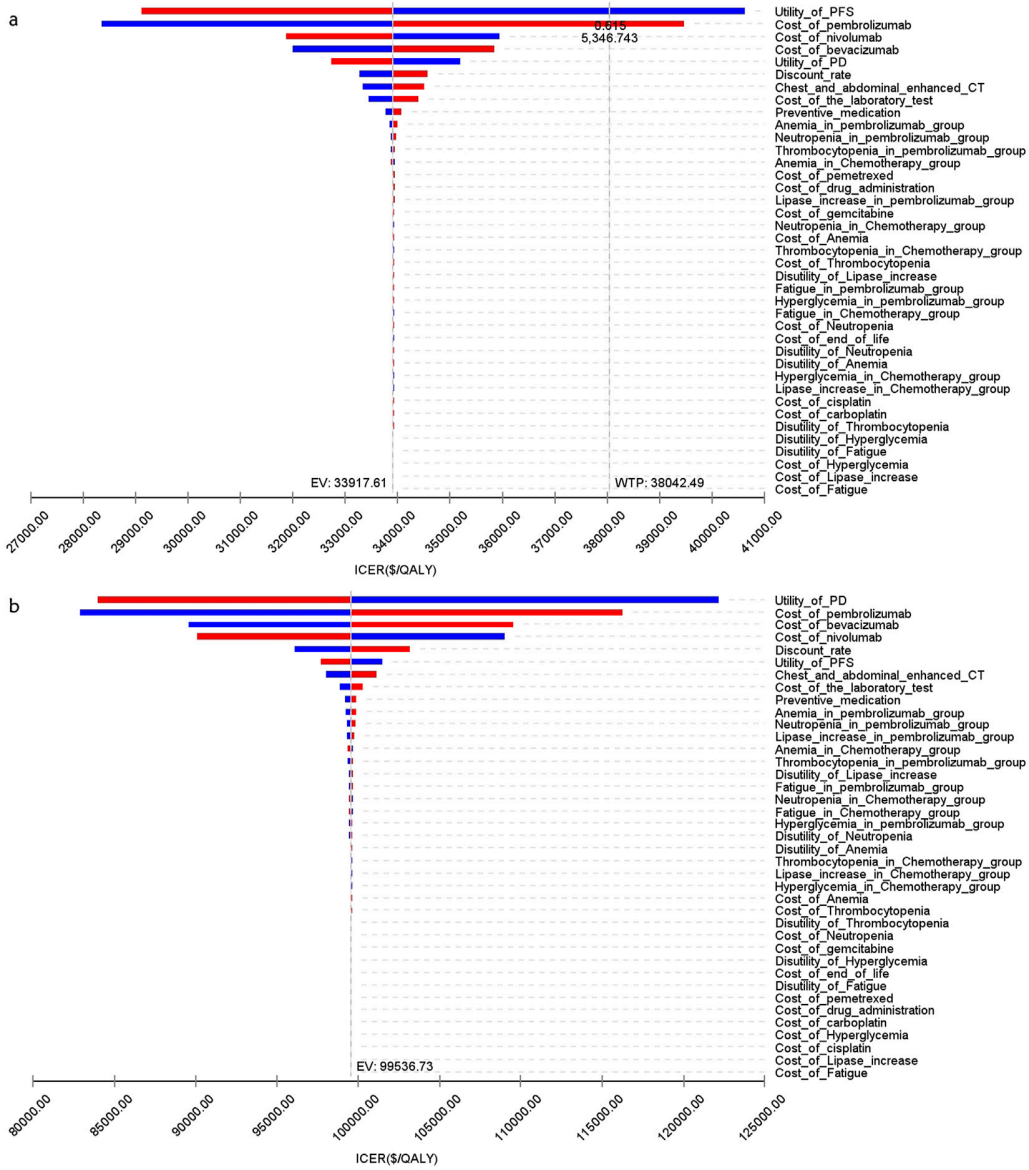
The tornado diagram of one-way sensitivity analysis **(A)** Patients with non-epithelioid group, **(B)** Patients with epithelioid group.

Figure 8 presents a tornado diagram from OWSA in the analysis that covers the entire population. This diagram shows the factors that exert the most significant influence on the base-case outcomes. The cost of pembrolizumab, the utility value of PD, and the cost of bevacizumab had the most pronounced impact on the base-case results. Figure 9 indicates that, for patients with non-epithelioid histology, ICER was mainly influenced by the utility of PFS, and the costs of pembrolizumab and nivolumab. In contrast, in patients with epithelioid histology, ICER was predominantly affected by three factors: the utility of PD, the cost of pembrolizumab, and the cost of bevacizumab, as shown in Figure 9. However, due to the marked differences in health outcomes between the two treatment strategies in all three groups, no variations in parameter values altered the study outcomes for these groups.

**FIGURE 10:**
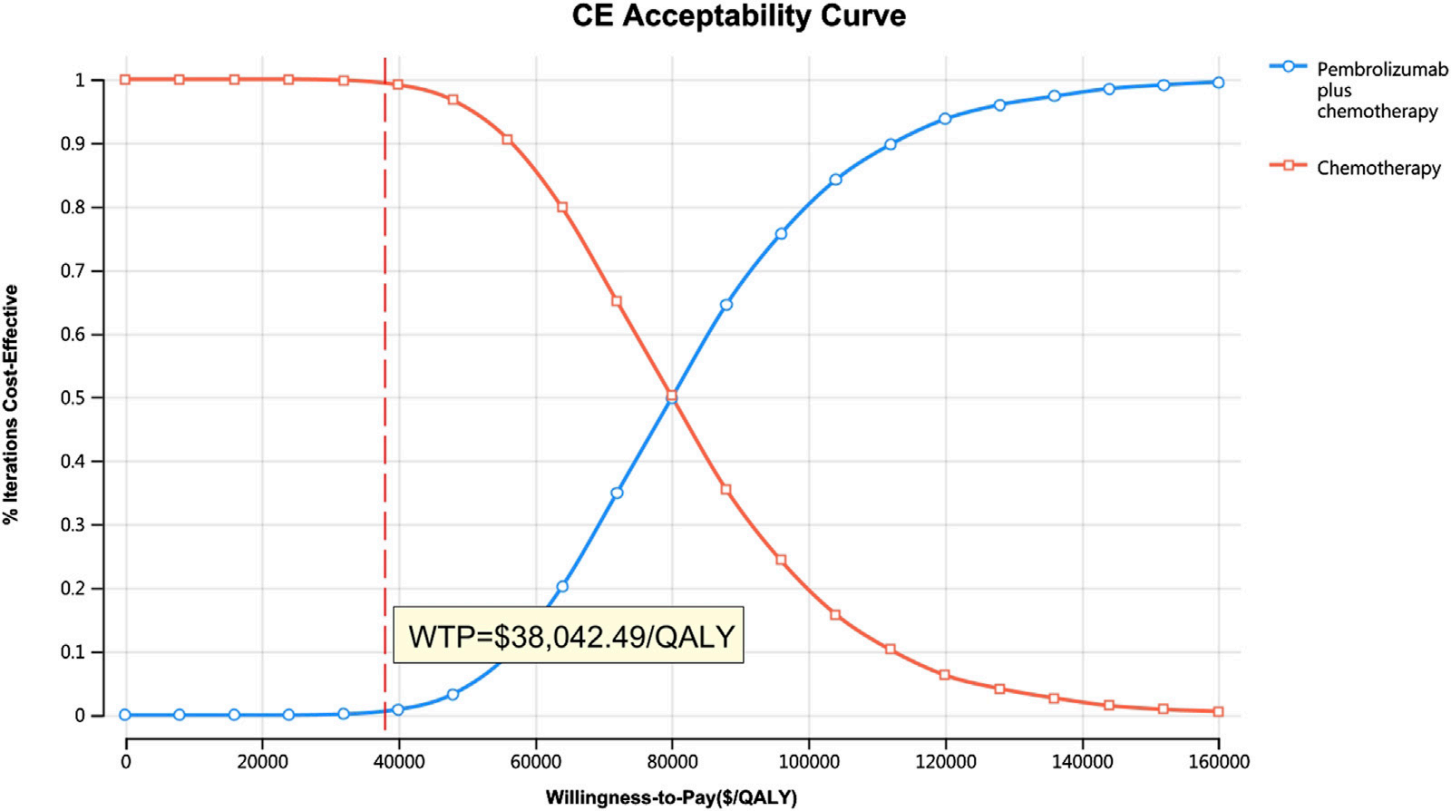
The cost-effectiveness acceptability curve.

**FIGURE 11:**
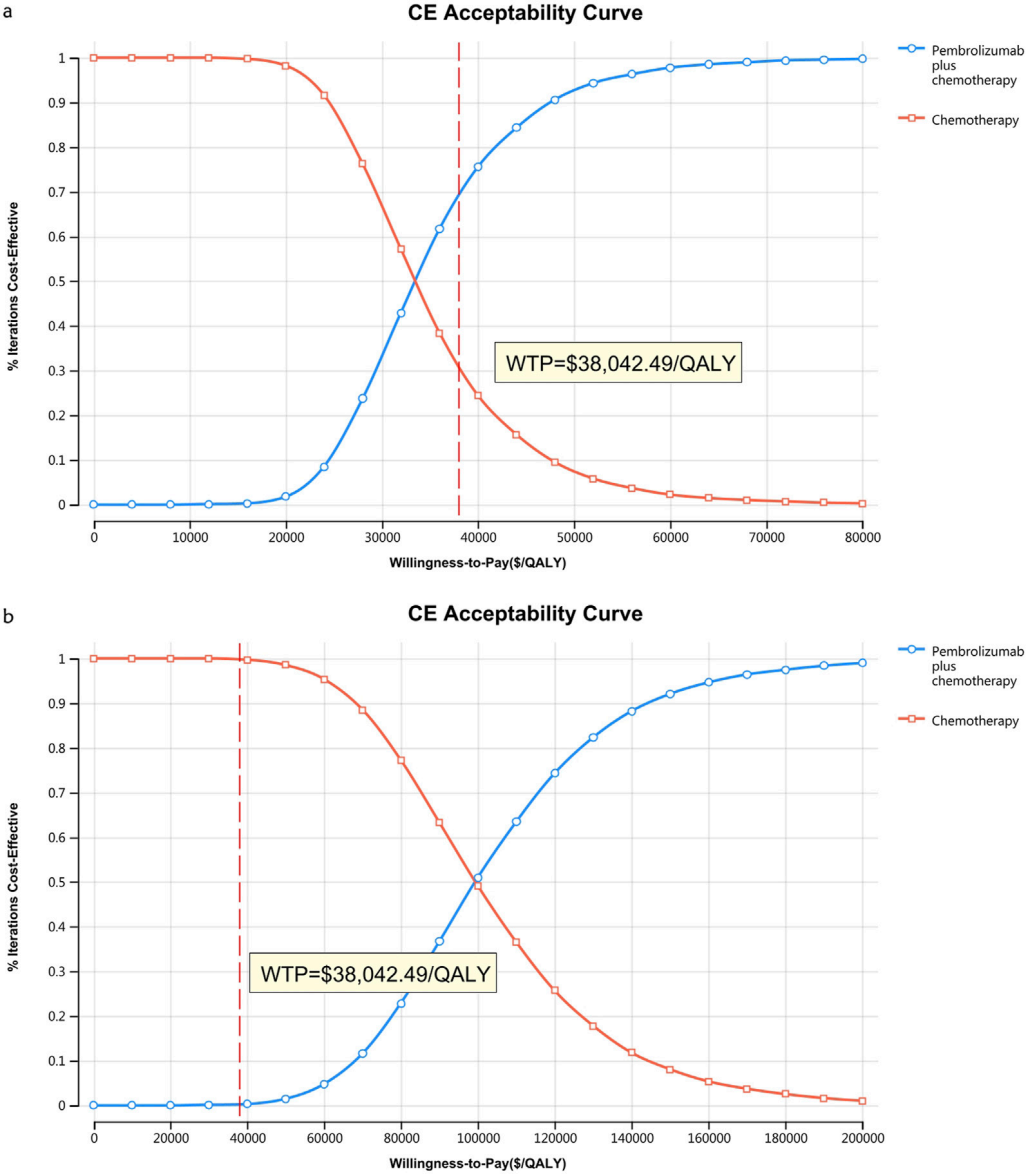
The cost-effectiveness acceptability curve **(B)** Patients with non-epithelioid group, **(B)** Patients with epithelioid group.

**FIGURE 12:**
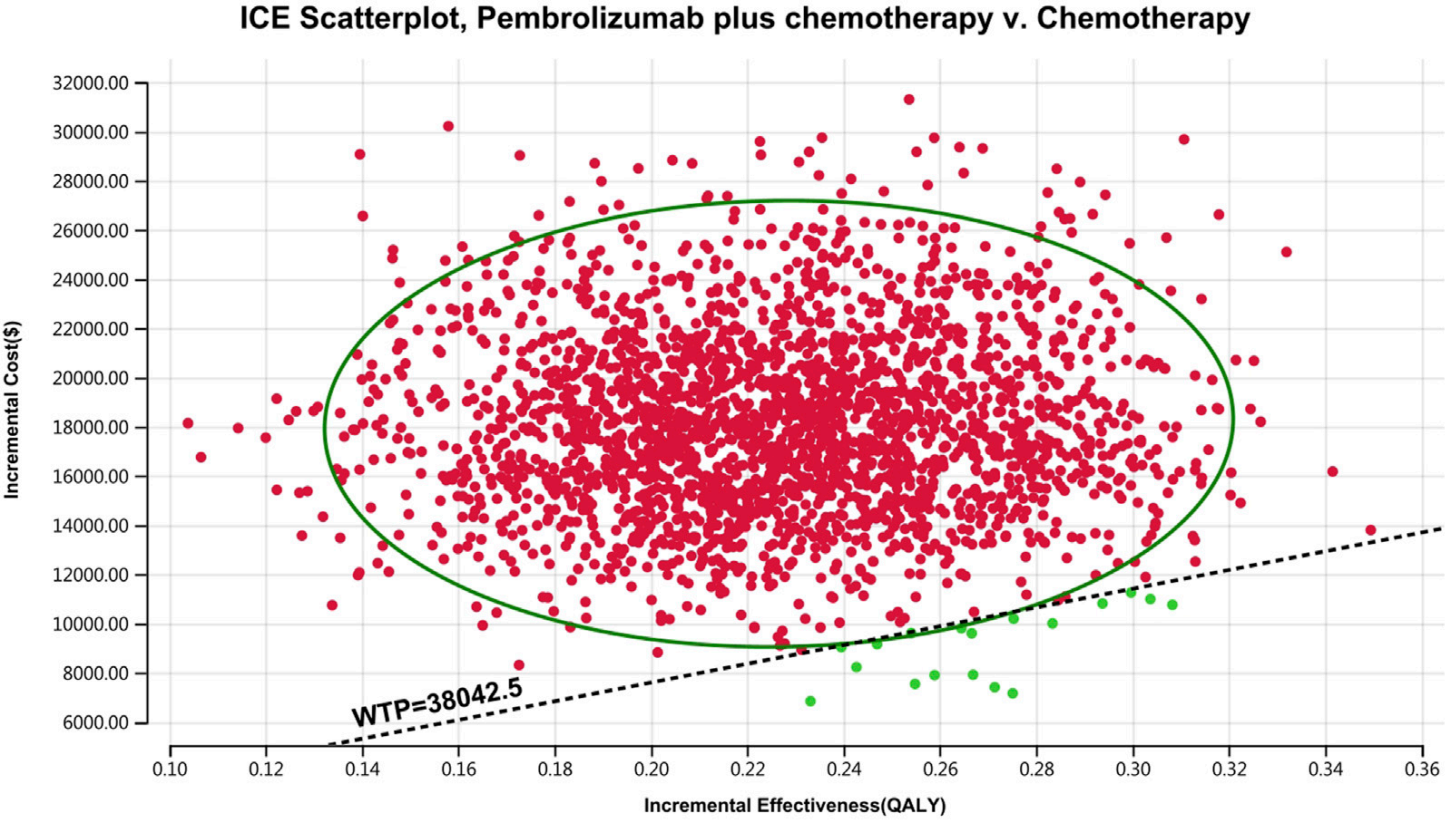
The cost-effectiveness probabilistic scatter plot.

**FIGURE 13:**
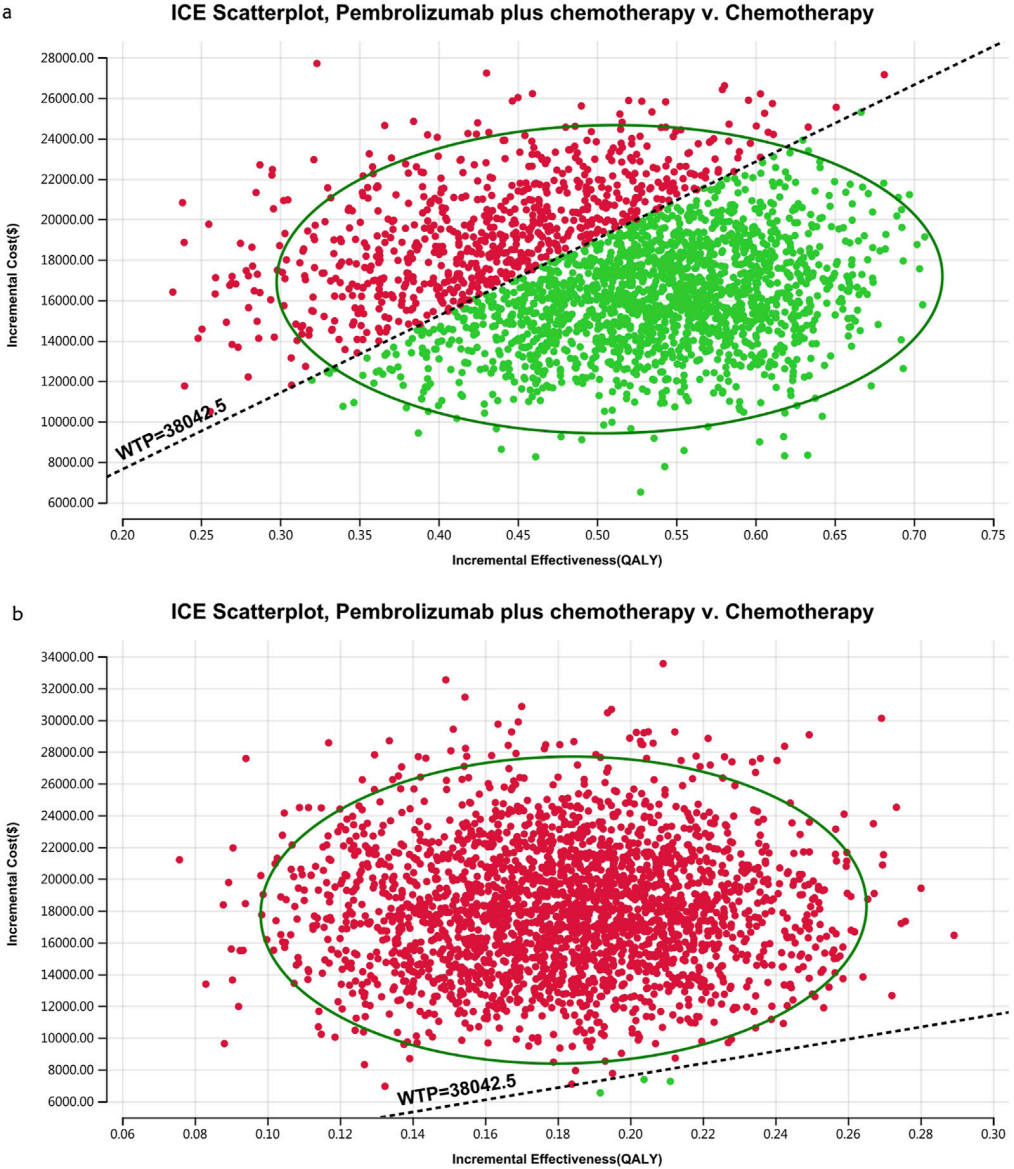
The cost-effectiveness probabilistic scatter plot **(A)** Patients with non-epithelioid group, **(B)** Patients with epithelioid group.

Figures 10–13 show acceptability curves and probabilistic scatter plots, which indicate the cost-effectiveness landscape. PSA outcomes revealed significant probabilities that pembrolizumab plus chemotherapy is deemed cost-effective. These probabilities were 0.55% for the entire population, 69.41% for those with non-epithelioid histology, and 0.14% for those with epithelioid histology, evaluated against a WTP threshold of three times China’s GDP *per capita* ($38,042.49).

## Discussion

Previous studies have suggested that immune checkpoint inhibitors are not cost-effective as first-line therapy in patients with advanced pleural mesothelioma (Yang L. et al., 2022; Ye et al., 2022; Barbier et al., 2023; Yang et al., 2023; Lang et al., 2024). However, the IND227 trial demonstrated that adding pembrolizumab to platinum and pemetrexed chemotherapy significantly improved the primary endpoint of overall survival and other key efficacy outcomes in these patients. In our study, compared to conventional treatment, pembrolizumab plus chemotherapy resulted in ICERs of $80,557.23 and $99,536.73 for the entire population and the epithelioid subgroup, respectively. However, in the non-epithelioid group, the ICER was $33,917.61. At current prices and the WTP threshold, pembrolizumab plus chemotherapy is more cost-effective than chemotherapy alone in advanced pleural mesothelioma. This assertion is supported by findings from INHB and INMB analyses, showing INHBs of −0.25, 0.05, and −0.29, and INMBs of $-9,605.00, $2,085.28, and $-11,127.42 in the respective patient subgroups compared to chemotherapy. Sensitivity analyses corroborated these base-case results.

However, no parameter value change altered the study results due to a significant health outcome gap between the two strategies in patients with the entire population and epithelioid histology. In contrast, for the non-epithelioid subgroup, with an ICER of $33,917.61/QALY, nearly every parameter influenced the study outcome, as this value is close to the WTP threshold of $38,042.49/QALY. The cost-effectiveness acceptability curve supports this, indicating a probability of 69.41% that this combination is cost-effective at a threshold of three times the GDP *per capita* per QALY. Therefore, reducing the prices of immune checkpoint inhibitors would benefit more patients.

In China, the National Healthcare Security Administration (NHSA) has engaged in multiple rounds of negotiations with pharmaceutical enterprises, predominantly focusing on the re-evaluation of the pricing structures of anticancer drugs. These negotiations have led to a significant reduction in the prices of various anticancer medications, thereby augmenting the cost-effectiveness of pembrolizumab from the perspective of the Chinese healthcare system. In the Chinese healthcare framework, the combination of pembrolizumab and chemotherapy was not more cost-effective than chemotherapy alone as an initial treatment for untreated advanced pleural mesothelioma, with the exception of patients with non-epithelioid histology. Hence, further validation of the cost-effectiveness of pembrolizumab plus chemotherapy would be requisite in the healthcare settings of developed countries, especially in the United States.

In the IND227 study, consistent with other research, patients with non-epithelioid histology had longer overall survival (Baas et al., 2021). The strengths of this study include being the first to evaluate the cost-effectiveness of pembrolizumab plus chemotherapy versus chemotherapy alone as first-line therapy for advanced pleural mesothelioma from the perspective of the Chinese health service system. It also uniquely assessed cost-effectiveness in the non-epithelioid and epithelioid subgroups, finding that immune checkpoint inhibitor plus chemotherapy is more cost-effective than chemotherapy alone in patients with non-epithelioid advanced pleural mesothelioma. The choices for second-line treatments are complex and varied, including surgery, chemotherapy, targeted therapy, immunotherapy, and radiation therapy. The IND227 trial was deficient in specific plans for second-line treatment, merely providing the utilization rate of second-line immunotherapy, which was three times higher in the chemotherapy group than in the pembrolizumab plus chemotherapy group. However, considering the Chinese context, the most pragmatic treatment plan was selected: bevacizumab plus gemcitabine plus carboplatin in the pembrolizumab plus chemotherapy group and nivolumab in the chemotherapy group. The one-way sensitivity analysis (OWSA) indicated that as the cost of second-line treatments in the pembrolizumab plus chemotherapy group escalated, the ICER also increased, while the converse was true in the chemotherapy group.

This study has several limitations. First, Quality of Life (EQ-5D) and cost per QALY data were not reported in the IND227 study, necessitating the use of utility values derived from the literature for PFS and PD, which could introduce uncertainties in the modeled results. Second, clinical data were obtained from a phase 3 trial conducted in Canada, Italy, and France, potentially introducing bias. Third, the lack of distinct data in the IND227 trial on the subsequent treatment plan, drug selection, and occurrence of AEs for non-epithelioid and epithelioid patients required assumptions to be made, assuming that these aspects were consistent with the general patient population. Despite these limitations, a cost-effectiveness analysis based on data from the IND227 study remains feasible and provides valuable information for treatment decision-making.

## Conclusion

In summary, this investigation demonstrates that pembrolizumab plus chemotherapy is a cost-effective therapy compared to chemotherapy for the first-line therapy of untreated advanced pleural mesothelioma patients with non-epithelioid histology from the Chinese healthcare system, but in entire population and those with non-epithelioid was not. The insights derived from this analysis provide a promising guide for decision-makers and medical professionals and evidence to support the broader application of pembrolizumab in clinical settings.

## Data Availability

The raw data supporting the conclusions of this article will be made available by the authors, without undue reservation.

## References

[B1] Baas P. Scherpereel A. Nowak A. K. Fujimoto N. Peters S. Tsao A. S. (2021). First-line nivolumab plus ipilimumab in unresectable malignant pleural mesothelioma (CheckMate 743): a multicentre, randomised, open-label, phase 3 trial. Lancet 397 (10272), 375–386. 10.1016/s0140-6736(20)32714-8 33485464

[B2] Barbier M. C. Fengler A. Pardo E. Bhadhuri A. Meier N. Gautschi O. (2023). Cost effectiveness and budget impact of nivolumab plus ipilimumab versus platinum plus pemetrexed (with and without bevacizumab) in patients with unresectable malignant pleural mesothelioma in Switzerland. Pharmacoeconomics 41 (12), 1641–1655. 10.1007/s40273-023-01305-3 37572261 PMC10635986

[B3] Chapel D. B. Stewart R. Furtado L. V. Husain A. N. Krausz T. Deftereos G. (2019). Tumor PD-L1 expression in malignant pleural and peritoneal mesothelioma by Dako PD-L1 22C3 pharmDx and Dako PD-L1 28-8 pharmDx assays. Hum. Pathol. 87, 11–17. 10.1016/j.humpath.2019.02.001 30794891

[B4] Chinese Pharmaceutical Association (2024). China pharmacoeconomic evaluation guideline 2020 (Draft for comments). Available at: https://www.cpa.org.cn/cpadmn/attached/file/20200929/1601363750111497.pdf (Accessed November 23, 2024).

[B5] Chu Q. Perrone F. Greillier L. Tu W. Piccirillo M. C. Grosso F. (2023). Pembrolizumab plus chemotherapy versus chemotherapy in untreated advanced pleural mesothelioma in Canada, Italy, and France: a phase 3, open-label, randomised controlled trial. Lancet 402 (10419), 2295–2306. 10.1016/s0140-6736(23)01613-6 37931632

[B6] Fennell D. A. Ewings S. Ottensmeier C. Califano R. Hanna G. G. Hill K. (2021). Nivolumab versus placebo in patients with relapsed malignant mesothelioma (CONFIRM): a multicentre, double-blind, randomised, phase 3 trial. Lancet Oncol. 22 (11), 1530–1540. 10.1016/s1470-2045(21)00471-x 34656227 PMC8560642

[B7] Freitag A. Sarri G. Ta A. Gurskyte L. Cherepanov D. Hernandez L. G. (2024). A systematic review of modeling approaches to evaluate treatments for relapsed refractory multiple myeloma: critical review and considerations for future health economic models. Pharmacoeconomics 42 (9), 955–1002. 10.1007/s40273-024-01399-3 38918342 PMC11343819

[B8] Guyot P. Ades A. E. Ouwens M. J. Welton N. J. (2012). Enhanced secondary analysis of survival data: reconstructing the data from published Kaplan-Meier survival curves. BMC Med. Res. Methodol. 12, 9. 10.1186/1471-2288-12-9 22297116 PMC3313891

[B9] Husereau D. Drummond M. Augustovski F. de Bekker-Grob E. Briggs A. H. Carswell C. (2022). Consolidated health economic evaluation reporting standards 2022 (CHEERS 2022) statement: updated reporting guidance for health economic evaluations. Value Health 25 (1), 3–9. 10.1016/j.jval.2021.11.1351 35031096

[B10] Lang W. Wei J. Jiang Q. Ai Q. Zhao X. Xiao L. (2024). Cost-effectiveness analysis of nivolumab versus placebo for relapsed malignant mesothelioma. Int. J. Clin. Pharm. 46 (1), 158–165. 10.1007/s11096-023-01662-1 37991664

[B11] Li W. Guo H. Li L. Cui J. (2021). Comprehensive comparison between adjuvant targeted therapy and chemotherapy for EGFR-mutant nsclc patients: a cost-effectiveness analysis. Front. Oncol. 11, 619376. 10.3389/fonc.2021.619376 33842322 PMC8027108

[B12] Lin Y. T. Chen Y. Liu T. X. Kuang F. Huang P. (2021). Cost-effectiveness analysis of camrelizumab immunotherapy versus docetaxel or irinotecan chemotherapy as second-line therapy for advanced or metastatic esophageal squamous cell carcinoma. Cancer Manag. Res. 13, 8219–8230. 10.2147/cmar.S335515 34754242 PMC8572144

[B13] Liu L. Wang L. Chen L. Ding Y. Zhang Q. Shu Y. (2023a). Cost-effectiveness of sintilimab plus chemotherapy versus chemotherapy alone as first-line treatment of locally advanced or metastatic oesophageal squamous cell carcinoma. Front. Immunol. 14, 1092385. 10.3389/fimmu.2023.1092385 36756110 PMC9899904

[B14] Liu S. Jiang N. Dou L. Li S. (2023b). Cost-effectiveness analysis of serplulimab plus chemotherapy in the first-line treatment for PD-L1-positive esophageal squamous cell carcinoma in China. Front. Immunol. 14, 1172242. 10.3389/fimmu.2023.1172242 37215110 PMC10192749

[B15] Mansfield A. S. Roden A. C. Peikert T. Sheinin Y. M. Harrington S. M. Krco C. J. (2014). B7-H1 expression in malignant pleural mesothelioma is associated with sarcomatoid histology and poor prognosis. J. Thorac. Oncol. 9 (7), 1036–1040. 10.1097/jto.0000000000000177 24926549 PMC4058651

[B16] Morris S. Gurusamy K. S. Patel N. Davidson B. R. (2014). Cost-effectiveness of early laparoscopic cholecystectomy for mild acute gallstone pancreatitis. Br. J. Surg. 101 (7), 828–835. 10.1002/bjs.9501 24756933

[B17] National Comprehensive Cancer Network (2024). Clinical practice guidelines in Oncology: mesothelioma pleural. Available at: www.nccn.org/patients.

[B18] Popat S. Baas P. Faivre-Finn C. Girard N. Nicholson A. G. Nowak A. K. (2022). Malignant pleural mesothelioma: ESMO Clinical Practice Guidelines for diagnosis, treatment and follow-up(☆). Ann. Oncol. 33 (2), 129–142. 10.1016/j.annonc.2021.11.005 34861373

[B19] Sahu R. K. Ruhi S. Jeppu A. K. Al-Goshae H. A. Syed A. Nagdev S. (2023). Malignant mesothelioma tumours: molecular pathogenesis, diagnosis, and therapies accompanying clinical studies. Front. Oncol. 13, 1204722. 10.3389/fonc.2023.1204722 37469419 PMC10353315

[B20] Scherpereel A. Antonia S. Bautista Y. Grossi F. Kowalski D. Zalcman G. (2022). First-line nivolumab plus ipilimumab versus chemotherapy for the treatment of unresectable malignant pleural mesothelioma: patient-reported outcomes in CheckMate 743. Lung Cancer 167, 8–16. 10.1016/j.lungcan.2022.03.012 35367910

[B21] Shu Y. Ding Y. Zhang Q. (2022). Cost-effectiveness of nivolumab plus chemotherapy vs. Chemotherapy as first-line treatment for advanced gastric cancer/gastroesophageal junction cancer/esophagel adenocarcinoma in China. Front. Oncol. 12, 851522. 10.3389/fonc.2022.851522 35515123 PMC9065445

[B22] Siegel R. L. Miller K. D. Fuchs H. E. Jemal A. (2021). Cancer statistics, 2021. CA Cancer J. Clin. 71 (1), 7–33. 10.3322/caac.21654 33433946

[B23] Sung H. Ferlay J. Siegel R. L. Laversanne M. Soerjomataram I. Jemal A. (2021). Global cancer statistics 2020: GLOBOCAN estimates of incidence and mortality worldwide for 36 cancers in 185 countries. CA Cancer J. Clin. 71 (3), 209–249. 10.3322/caac.21660 33538338

[B24] Vogelzang N. J. Rusthoven J. J. Symanowski J. Denham C. Kaukel E. Ruffie P. (2003). Phase III study of pemetrexed in combination with cisplatin versus cisplatin alone in patients with malignant pleural mesothelioma. J. Clin. Oncol. 21 (14), 2636–2644. 10.1200/jco.2003.11.136 12860938

[B25] Wang Q. Xu C. Wang W. Zhang Y. Li Z. Song Z. (2023). Chinese expert consensus on the diagnosis and treatment of malignant pleural mesothelioma. Thorac. Cancer 14 (26), 2715–2731. 10.1111/1759-7714.15022 37461124 PMC10493492

[B26] Wang S. Khan F. I. (2023). Investigation of molecular interactions mechanism of pembrolizumab and PD-1. Int. J. Mol. Sci. 24 (13), 10684. 10.3390/ijms241310684 37445859 PMC10341962

[B27] Wei Q. Liang Y. Mao J. Guan X. (2024). Cost-effectiveness analysis of adjuvant alectinib versus platinum-based chemotherapy in resected ALK-positive non-small-cell lung cancer in the Chinese health care system. Cancer Med. 13 (22), e70405. 10.1002/cam4.70405 39555835 PMC11571239

[B28] Wu B. Li T. Cai J. Xu Y. Zhao G. (2014). Cost-effectiveness analysis of adjuvant chemotherapies in patients presenting with gastric cancer after D2 gastrectomy. BMC Cancer 14, 984. 10.1186/1471-2407-14-984 25526802 PMC4301844

[B29] Wu Q. Liao W. Zhang M. Huang J. Zhang P. Li Q. (2020). Cost-effectiveness of tucatinib in human epidermal growth factor receptor 2-positive metastatic breast cancer from the US and Chinese perspectives. Front. Oncol. 10, 1336. 10.3389/fonc.2020.01336 32850425 PMC7417356

[B30] Yang J. Han J. Zhang Y. Muhetaer M. Chen N. Yan X. (2022a). Cost-effectiveness analysis of trastuzumab deruxtecan versus trastuzumab emtansine for HER2-positive breast cancer. Front. Pharmacol. 13, 924126. 10.3389/fphar.2022.924126 36160459 PMC9500475

[B31] Yang L. Cao X. Li N. Zheng B. Liu M. Cai H. (2022b). Cost-effectiveness analysis of nivolumab plus ipilimumab versus chemotherapy as the first-line treatment for unresectable malignant pleural mesothelioma. Ther. Adv. Med. Oncol. 14, 17588359221116604. 10.1177/17588359221116604 35958872 PMC9358333

[B32] Yang L. Song X. Zeng W. Zheng Z. Lin W. (2023). First-line nivolumab plus ipilimumab for unresectable MPM in China: a cost-effectiveness analysis. Orphanet J. Rare Dis. 18 (1), 326. 10.1186/s13023-023-02925-w 37845696 PMC10580582

[B33] Yap T. A. Aerts J. G. Popat S. Fennell D. A. (2017). Novel insights into mesothelioma biology and implications for therapy. Nat. Rev. Cancer 17 (8), 475–488. 10.1038/nrc.2017.42 28740119

[B34] Ye Z. M. Tang Z. Q. Xu Z. Zhou Q. Li H. (2022). Cost-effectiveness of nivolumab plus ipilimumab as first-line treatment for American patients with unresectable malignant pleural mesothelioma. Front. Public Health 10, 947375. 10.3389/fpubh.2022.947375 35937220 PMC9354521

[B35] Zhan M. Zheng H. Xu T. Yang Y. Li Q. (2017). Cost-effectiveness analysis of additional bevacizumab to pemetrexed plus cisplatin for malignant pleural mesothelioma based on the MAPS trial. Lung Cancer 110, 1–6. 10.1016/j.lungcan.2017.05.012 28676211

[B36] Zhu Y. Liu K. Wang M. Wang K. Zhu H. (2022). Trastuzumab deruxtecan versus trastuzumab emtansine for patients with human epidermal growth factor receptor 2-positive metastatic breast cancer: a cost-effectiveness analysis. Breast 66, 191–198. 10.1016/j.breast.2022.10.010 36327624 PMC9619174

[B37] Zhu Y. Liu K. Zhu H. Wu H. (2023). Immune checkpoint inhibitors plus chemotherapy for HER2-negative advanced gastric/gastroesophageal junction cancer: a cost-effectiveness analysis. Ther. Adv. Gastroenterol. 16, 17562848231207200. 10.1177/17562848231207200 PMC10624011 37928895

